# Decisional Balance Inventory (DBI) Adolescent Form for Smoking: Psychometric Properties of the Persian Version

**DOI:** 10.1186/s12889-017-4425-2

**Published:** 2017-05-25

**Authors:** Maryam Khazaee-Pool, Tahereh Pashaei, Ponnet Koen, Fatemeh Jafari, Rashin Alizadeh

**Affiliations:** 1 Department of Health Education and Promotion, School of Public Health, Zanjan University of Medical Sciences, Zanjan, Iran; 2 Department of Public Health, School of Health, Kurdistan University of Medical Sciences; Social Determinants of Health Research Center, Kurdistan University of Medical Sciences, Sanandaj, Iran; 3 Department of Communication Sciences, Ghent University, Ghent, Belgium; 4 Department of Communication Sciences, University of Antwerp, Antwerp, Belgium; 5 Department of Public Health, School of Public Health, Zanjan University of Medical Sciences, Zanjan, Iran; 6 Department of Health Education and Promotion, School of Health, Tehran University of Medical Sciences, Tehran, Iran

**Keywords:** Decisional balance inventory, Reliability, Validity, Smokers

## Abstract

**Background:**

One effective model for studying cigarette smoking cessation is the transtheoretical model (TTM). In order to assess to what degree interventions can make variations in individuals’ behavior, several questionnaires have been developed based on the TTM. This study aims to describe the development of the Persian version of the Decisional Balance Inventory (DBI) for smoking cessation in Iran and to evaluate its psychometric properties.

**Design and methods:**

The forward-backward technique was used to translate the DBI from English into Persian. After linguistic validation and a pilot test among 30 male smoking young adults, a cross-sectional study was performed, and psychometric properties of the Persian version of the DBI were assessed. Using a convenience sampling method, 120 male smokers between 16 and 24 years of age were recruited from three factories in Nowshahr, Iran. In order to assess the reliability of the DBI, internal consistency and test–retest methods were performed. Additionally, face and content validity were assessed, and the construct validity of the DBI was calculated by performing both exploratory and confirmatory factor analysis. Data were analyzed using SPSS and AMOS.

**Results:**

The mean age of the sample (*n* = 120) was 20.19 (SD = 2.13) years. The mean scores for the content validity index (CVI) and the content validity ratio (CVR) were .94 and .89, respectively. The results of exploratory factor analysis (EFA) showed a three-factor solution for the DBI that accounted for 55.4% of observed variance. The results achieved from the confirmatory factor analysis (CFA) displayed that the data fit the model: the relative chi-square (×2/df) = 1.733 (*p* < .001) and the root mean square error of approximation (RMSEA) = .07 (90% CI = .05–.105). All comparative indices of the model including GFI, AGFI, CFI, NNFI, and NFI were more than .80 (.87, .83, .91, .89, and .81, respectively). The Cronbach’s alpha ranged from .78 to .83, indicating an acceptable reliability. Furthermore, the intraclass correlation coefficient (ICC) ranged from .72 to .89, confirming a satisfactory result.

**Conclusions:**

The results from the present study indicate that the Persian version of the DBI has good psychometric properties and is suitable to measure smoking behaviors among Iranian adolescent and young adult smokers. Consequently, the instrument could be used in planning cigarette smoking cessation interventions among Iranian adolescents and young adults.

## Background

One preventable cause of early mortality is cigarette smoking [1]. Although the tobacco smoking rate is reducing overall, more than two-thirds of recent deaths in developing countries are caused by diseases related to smoking tobacco. Based on a report of the World Health Organization (WHO), 22% of the world’s population aged 15 years and above are smokers, and almost 6 million people die from exposure to tobacco smoke or from tobacco use [2]. According to a study from 2012, the prevalence rate of current daily smoking in Iran is respectively 11.3% (21.4% of men and 1.4% of women) and 12.5% (23.4% of men and 1.4% of women). Furthermore, it was reported that the mean number of cigarettes smoked every day by Iranian smokers was 13.7 sticks [1].

The large number of population still use cigarette smoking in spite of its risky impacts. It is recognized that quitting cigarette smoking results in several health benefits, such as reduced mortality risk due to cardiovascular diseases [3, 4]. Because of the strongly addictive nature of nicotine, relapse after stopping is common. Furthermore, there are some social benefits of smoking that prevent smokers from quitting, like enhanced feelings of relaxation and a sense of control [5–7]. Persons who smoke for a long time are commonly not influenced by the long-term benefits of quitting cigarette smoking, especially when the diseases that are associated with smoking have not developed obviously [8–10].

Interpersonal communication has an important effect on smoking. From the perspective of social cognition, the decision of whether or not to smoke is influenced by the response of peers in one’s social environment. For instance, individuals may be discouraged from smoking cigarettes if they believe that smoking is perceived as a negative behavior by the public and that they may face social disapproval by doing so. As such, the negative social consequences to smoking, such as peer refusal, are closely associated with a decreased probability of continued smoking [11, 12]. Thus, individuals who received negative consequences may have less of a tendency to smoke cigarettes [13].

Successful approaches to the cessation of cigarette smoking are often based on behavioral change models [14]. The transtheoretical model (TTM) is known as one of the most important models in the field of preventive health behavior [15, 16]. This model, developed by Prochaska, assesses an individual’s readiness to act on a new, healthier behavior and provides approaches for change to guide the individual [17]. The TTM is composed of four constructs: stages of change, processes of change, self-efficacy, and decisional balance and temptations [18–20]. The TTM is based on the assumption that people are at different stages of motivational readiness for engaging in healthier behaviors and that intervention approaches are most effective when they are matched to a person’s current stage of change [15, 21]. Decisional balance, which is the focus of the present article, refers to the idea that pros and cons are important in the decision-making process for behavioral change.

Based on the TTM, several scales were developed to assess to what degree interventions can cause variations in individuals’ behavior. One of the TTM-based scales that measure lifestyle changes like smoking is the Decisional Balance Inventory (DBI), which measures positive thoughts (pros) and negative thoughts (cons) that might occur to an individual who is deciding whether or not to smoke [22]. The initial scale comprised 24 items, but later on, Pallonen et al. (1998) developed a DBI with 12 items [23]. In developed countries, the English version of the DBI has been validated in many studies [24–27], but unfortunately, there is limited literature on this topic in developing countries like Iran .

There is need for a questionnaire on health behavior changes that can be applied in various cultural settings. Culturally and linguistically competent scales like the DBI consider cultural values, beliefs, and practices that vary among diverse people. So, it is essential to re-assess the validity and reliability of this scale in a specific culture, like the Iranian one. Therefore, the aim of this study is to examine the psychometric properties of the Persian version of the DBI for smoking cessation in Iranian adolescents and young adults. Consistent with other studies, albeit in a different context [24–27], we expect the Persian version of the DBI to have good psychometric properties.

## Methods

### The Decisional Balance Inventory (DBI)

The DBI is a self-report instrument that focuses on either a positive thought (pro) or a negative thought (con) that might happen to a person who is deciding whether or not to smoke or not smoke. The original DBI was developed by Velicer and comprises 24 items that assess the opinions of adolescents about the damages and benefits of smoking [22]. The brief DBI was developed by Pallonen in 1998 and consists of 12 items. The shortened DBI measures one of the key constructs of the TTM and consists of three factors, including cons of smoking (six items), social pros (three items) and coping pros (three items), and each item is rated on a five-point Likert-type scale (1 = *not important* to 5 = *extremely important*) [23].

### Translation

After getting permission from the author, the forward-backward method was used to translate the DBI from English into Persian. For the forward translation, two independent expert translators translated the scale into Persian. Then, the Persian versions were compared by one of the authors and both of the translators, and they made a single temporary Persian version of the DBI. For the backward step, two other English professionals translated the temporary Persian version of the DBI back into English, and a temporary English version was made. Both translators were fluent in both Persian and English, and they were also skilled health care experts who have been employed for many years. In the next step, the study team and the translators tested the scale for accuracy. In order to measure the content validity of the DBI, an expert panel including seven health professionals (two psychologists, two health education experts, one epidemiologist, and two specialists in tobacco control) compared the provisional English version of the DBI with the original scale, and after some verbal and cultural adaptations, the pre-final Persian version of the DBI was made. This version was assessed in a pilot study with 30 male smoking adolescents. Eventually, the final Persian version of the DBI was produced, and it was used in this study [28].

## Design and data collection

A cross-sectional validation study was carried out in Nowshahr, Iran. A convenient sample of male smokers between 16 and 24 years of age who worked in three factories in Nowshahr, Mazandaran participated in the study. The inclusions criteria were (a) being a current smoker who has smoked at least 100 cigarettes total, (b) not having participated in any effort to quit, (c) planning to quit smoking in the next 30 days, and (d) having the ability to read and write in Farsi. We recruited individuals who were in this stage to quit smoking because we were planning to perform an intervention for such a group. According to the TTM, every stage of change needs its own approach to ensure a successful intervention [18]. Therefore, we believed that the best suited sample for this study would be a sample of participants who were in a preparation stage to quit smoking. Otherwise, we would have had to create a number of different interventions for the individuals in various stages of change, that would have been difficult due to limited time and resources.

The sample size was estimated a priori. The sample size was determined based on the number of items in the scale multiplied by 10 (12 × 10 = 120) [29]. A convenience sampling method was applied to recruit the respondents. An introductory letter was sent to four factories through personal contacts of the researchers. A positive reply to cooperate on this study was received from three factories. Formal consent from the factories’ manager and participants was required prior to the study. We asked the managers of the selected factories for permission to perform a study in their factory. The self-administered paper-and-pencil questionnaire was conducted during work time in the presence of a researcher, who explained the purpose and procedure of the study. Participants were assured their answers were anonymous and confidential, and that they could leave the study at any given time that they want. Afterwards, participants informed consent was achieved. The questionnaire took 25–30 min to complete.

## Statistical analysis

Psychometric properties of the Persian version of the DBI were measured by the following statistical analyses:

## Validity

Content, face and construct validity of the Persian version of the DBI were measured.

### Content validity

Both qualitative and quantitative methods were used to assess content validity. In the qualitative stage, an expert panel consisting of seven health experts, including two psychologists, two health education experts, one epidemiologist, and two specialists in tobacco control, assessed the content validity. The experts assessed wording, grammar, item allocation and scaling of the DBI. The content validity index (CVI) and the content validity ratio (CVR) were calculated in the quantitative stage. CVI measures the relevancy, clarity, and simplicity of each item [30, 31]. In order to calculate the CVI, a Likert-type ordinal scale with four possible responses was applied. The answers were rated from 1 = *not relevant, not simple and not clear* to 4 = *very relevant, very simple and very clear*. The CVI was assessed as the proportion of items on a scale that attained a rating of 3 or 4 by the experts [32]. The CVR tested the essentiality of each item in a scale. In order to assess the CVR, the experts rated each item as 1 = essential, 2 = useful but not essential, or 3 = not essential. Then, based on the Lawshe Table, items with a CVR score of 0.62 or above were considered to be acceptable and were retained [33].

### Face validity

To assess the face validity, both qualitative and quantitative methods were used. A group of smoking male young adults (*n* = 10) were asked to evaluate each item of the scale and to indicate if they felt difficulty or ambiguity in replying to the Persian version of the DBI. Thereafter, the impact score (frequency × importance) was assessed to show the percentage of smoker men who identified each item as important or quite important on a five-point Likert scale. Items were considered to be appropriate if they had an impact score equal to or more than 1.5 (which corresponds to a mean frequency of 50% and a mean importance of three on the five-point Likert scale) [34].

### Construct validity

Confirmatory factor analysis (CFA) was applied in order to assess the coherence between the data and the structure. The model fit was evaluated using multiple fit indices. As suggested, various fit indices measuring relative Chi-square, Goodness of Fit Index (GFI), Comparative Fit Index (CFI), Root Mean Square Error of Approximation (RMSEA), Non-Normed Fit Index (NNFI), Normed Fit Index (NFI) and Standardized Root Mean Square Residual (SRMR) were taken into account [35, 36]. The GFI, CFI, NFI, and NNFI value range between 0 and 1 [37], but values equal to .80 or above are commonly indicated as acceptable model fits [37]. An RMSEA value between .08 and .10 demonstrates an average fit, and a value below .08 shows a good fit. Values below .05 indicate a good fit for SRMR, but values between .05 and .08, and between .08 and .10 indicate a close fit or are acceptable, respectively [38].

## Reliability

In order to assess the reliability of the DBI, the internal consistency was tested applying the Cronbach’s alpha coefficient. The alpha values equal to .70 or higher were considered acceptable [30]. Furthermore, intraclass correlation coefficient (ICC) was estimated for assessing the stability of DBI. The scale was re-administered to 40 smokers below 25 years of age 1 week after the first completion. ICC values of .40 or above are considered acceptable (r’s between .81 and 1.0 are excellent, between 0.61 and .80 are very good, between .41 and .60 are good, between .21 and .40 are fair, and between .0 and .20 are poor) [29]. The analyses were performed using the statistical program SPSS for Windows version 23.0 and Amos 24.0.

## Results

### The study sample

A total of 142 male smokers between 16 and 24 years of age completed the DBI. We excluded 22 questionnaires because they did not provide complete demographic information, resulting in 120 participants for analyses. The mean age of the participants was 20.19 years (SD = 2.13). About 27% (32 participants) had primary education, 62% (75 participants) had secondary education, and 11% (13 participants) had higher education. The most common age to start smoking was 13–15 years (50.8% participants). About 30% (36 participants) said that they started smoking between 16 and 20 years of age, and 13.3% (16 participants) started smoking before 12 years of age [see Table 1].

**Table 1:** Descriptive characteristics of the study sample (*n* = 120)

	Number	%
Age (years)		
≤ 18	26	21.7
19–21	56	46.7
22–24	38	31.6
Mean (SD)	20.19 (2.13)	
Range	16–24	
Marital status		
Single/divorced	102	85.0
Married	18	15.0
Educational Level		
Primary	32	26.7
Secondary	75	62.5
Higher	13	10.8
Age of beginning to smoke cigarettes		
≤ 12	16	13.3
13–15	61	50.8
16–20	36	30.0
20–24	7	5.8

### Validity

An EFA was applied on the 12 items of the DBI (cut-off point: .50). Factor loadings of each item and the three subscales are presented in Table 2. All items loaded on their respective construct. The three constructs of the DBI jointly accounted for 55.4% of the observed variance.

**Table 2:** Exploratory factory analysis of the DBI (*n* = 120)

Item	Factor 1	Factor 2	Factor 3
6. Smoking can affect the health of others	**.753**	.041	.094
9. Smoking cigarettes is hazardous to people’s health	**.735**	.146	−.234
3. Smoking stinks	**.676**	−.012	.176
11. Smoking is a messy habit	**.659**	.269	.249
10. Cigarette smoking bothers other people	**.617**	.074	.022
12. Smoking makes teeth yellow	**.596**	.274	.080
1. Smoking makes kids get more respect from others	.182	**.890**	.055
4. Kids who smoke have more friends	.145	**.887**	−.004
7. Kids who smoke go out on more dates	.080	**.605**	.115
5. Smoking cigarettes is pleasurable	.000	−.039	**.808**
2. Smoking helps people to cope better with frustrations	.233	.171	**.648**
8. Smoking cigarettes relieves tension	.018	.064	**.624**

*Note.* Figures in bold relate to factor loadings equal to or higher than .50

**Fig. 1:**
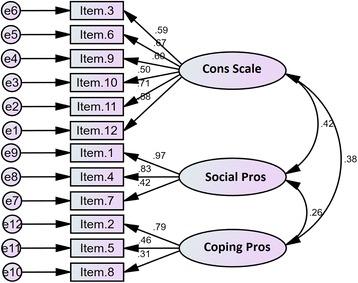
A three-factor model for the DBI obtained from CFA (*n* = 120)

We conducted a CFA on the 12-item questionnaire to test the fitness of the model obtained from the EFA. Fig. 1 shows the best model fit. Covariance matrixes were used and fit indexes were calculated. All fit indices proved to be good. The relative chi-square (χ2/df) was equal to 1.733 (*p* < .001). The RMSEA of the model was .07 (90% CI = .05–.105), and the SRMR was .07. All comparative indices of the model, including GFI, AGFI, CFI, NNFI, and NFI, were more than .80 (.87, .83, .91, .89, and .81, respectively).

Although the model fitted good, modification indices for the regression weights were examined for identifying covariance among the factors. No significant improvement on fit indexes was obtained, so no changes were made and the model was accepted in its present form. Fig. 1 shows the model.

### Reliability

To measure the internal consistency, the Cronbach’s alpha was calculated separately for the DBI as well as for each factor of the DBI. The Cronbach’s alpha coefficient for the DBI was .92 and ranged from .78 to .83 for its subscales, which is well above the acceptable threshold. Furthermore, test-retest analysis was conducted to test the stability of the DBI. The results indicated satisfactory results. Intra-class correlation (ICC) was .93 for the DBI and ranged from .72 to .89 (good to excellent) for the subscales of the DBI, lending support for the stability of the questionnaire. The results are presented in Table 3.

**Table 3:** Measures of the internal consistency and intraclass coefficient

Factor	The name of factor	Number of items	Cronbach’s alpha	ICC
1	Cons Scale	6 items (3, 11, 12, 9, 10, 6)	0.800	0.896
2	Social Pros	3 items (1, 4, 7)	0.787	0.724
3	Coping Pros	3 items (5, 2, 8)	0.832	0.813
Total		12 items	0.928	0.933

## Discussion

The aim of the current study was to perform a psychometric evaluation for the translated Persian version of an additional component of the TTM, the Decisional Inventory Index (DBI) for smoking cessation. This scale measures movement through the stages of change and delivers insight into mechanisms through which individuals try to change their risky behaviors. In general, the results demonstrated that the translated DBI is a suitable and valid questionnaire that can be used for assessing smoking behavior among adolescent and young adult smokers who speak Persian. Developing theory-based questionnaires can be considered a main precondition for the evaluation of any intervention program. Therefore, we consider the results from this study to be useful for adolescent and young adult who are part of a cigarette smoking control plan.

The Cronbach’s alpha and the ICC were acceptable and showed good reliability and stability for the DBI. Furthermore, the CVI and the CVR showed a reasonable content validity. The EFA results were consistent with those found by the original developer of the DBI. This indicates that the DBI is effective for presenting multiple aspects of the health concerns affected by smoking. These findings are also similar to studies that have been conducted by other investigators in other contexts [39, 40].

As expected, the present study showed a three-factor solution for the Persian version of the DBI, including social pros as well as coping pros and cons. The three factors were able to predict 55.4% of the observed variance. This result is somewhat higher than that found by Velicier (1985), in whose study a two-factor solution (i.e., pros and cons) accounted for 41% of the observed variance [22], and it is also higher than that found by Pallonen et al. (1998), in whose study a three-factor solution (i.e., social pros, coping pros, and cons) accounted for 50% of the total variance [23]. In another study among both smokers and nonsmokers by Hoeppner et al. (2012), a four-factor solution (i.e., two pro factors, two con factors) was obtained that explained 45% of the variance [41].

The results also demonstrated that the questionnaire is able to discriminate between the perceived benefits and barriers involved in making the decision to quit smoking. Decisional balance is a key construct of the TTM, and the results of the DBI imply that pros and cons are comparatively significant parts of the model. With regard to changing health and risk behaviors among target groups, it is important to emphasize the pros and cons for that specific behavior.

We also performed the CFA to determine if there was coherence between the data and the theoretical structure. The CFA provided good fit indices for the present model, and the convergent validity of the subscales of the DBI was acceptable. These findings are consistent with studies conducted in different cultural backgrounds [22, 23, 42], which have indicated that the DBI is reliable when it is applied in Persian-speaking smokers. Our findings also demonstrated that the model from the original scale is similar to our model [23]. Furthermore, the internal consistency of the scale as measured by the Cronbach’s alpha revealed an acceptable reliability for all subscales, which was consistent with previous studies [22, 23]. Furthermore, after examining 40 male young adult smokers over a one-week period, our findings clearly indicated that the DBI has good stability in the short term; however, it has yet to be seen whether the DBI is still stable in the long term.

## Limitations

The present study has also some limitations. One limitation has to do with the accuracy of the participants’ answers, because all measures were self-reported. Another limitation of the current study is related to its generalizability and its sample size. The present sample was limited to a convenient sample of 120 male adolescent and young adult smokers, and it is unknown whether we would achieve the same outcomes if a large representative group of both male and female smokers were recruited. As such, the current study is unable to assess gender differences with regard to the psychometric properties of the DBI. Future studies should aim to include both male and female smokers in order to assess whether motivations for quitting smoking are similar between the genders and whether gender influences amenability to treatment. Furthermore, this study included only adolescents and young adults who were working in a factory, thus excluding students. Future studies should also assess the psychometric properties of the Persian version of the DBI in an Iranian adolescent and young adult student sample. Finally, the sample of the current study was ethnically homogenous (just Farsi); further studies need to consider the relationship between different Iranian ethnicities (e.g., Gilak, Turkish, Kurdish, Baluchi, and so on) and the DBI.

## Conclusion

The findings suggest that the Persian version of the DBI is a reliable and valid scale to determine smoking behaviors among Iranian male smokers. As such, the Persian version of DBI may be supportive for healthcare teams to identify and to design health approaches that are practical and targeted to specific situations. Further studies in male and female populations are recommended to establish stronger psychometric properties for the DBI.

## Abbreviations

CFA: Confirmatory factor analysis
CFI: Comparative fit index
Cons: Negative thoughts
CVI: Content validity index
CVR: Content validity ratio
DBI: Decisional blance inventory
EFA: Exploratory factor analysis
GFI: Goodness of fit index
ICC: Intraclass correlation coefficient.
NFI: Normed fit index
NNFI: Non-Normed fit index
Pros: Positive thoughts
RMSEA: Root mean square error of approximation
SRMR: Standardized root mean square residual
TTM: Transtheoretical model
