# Correction to: Decisional Balance Inventory (DBI) Adolescent Form for Smoking: Psychometric Properties of the Persian Version

**DOI:** 10.1186/s12889-017-4927-y

**Published:** 2017-11-30

**Authors:** Maryam Khazaee-Pool, Tahereh Pashaei, Koen Ponnet, Fatemeh Jafari, Rashin Alizadeh

**Affiliations:** 1 Department of Health Education and Promotion, School of Public Health, Zanjan University of Medical Sciences, Zanjan, Iran; 2 Social Determinants of Health Research Center, Kurdistan University of Medical Sciences, Sanandaj, Iran; 3 Department of Communication Sciences, Ghent University, Ghent, Belgium; 4 Department of Communication Sciences, University of Antwerp, Antwerp, Belgium; 5 Department of Public Health, School of Public Health, Zanjan University of Medical Sciences, Zanjan, Iran; 6 Department of Health Education and Promotion, School of Health, Tehran University of Medical Sciences, Tehran, Iran; 7 Department of Public Health, Faculty of Health, Kurdistan University of Medical Sciences, Sanandaj, Iran

## Correction

After publication of the article [1], it has been brought to our attention that the first and last names of the third author were transposed in the original article. The author was published as “Ponnet Koen” where in fact the correct name is “Koen Ponnet”. The original article has been revised to reflect this.

## References

[1] Khazaee-Pool M, Pashaei T, Ponnet K, Jafari F, Alizadeh R. Decisional balance inventory (DBI) adolescent form for smoking: psychometric properties of the Persian version. BMC Public Health. 2017;17(1) 10.1186/s12889-017-4425-2. 10.1186/s12889-017-4425-2 PMC5445340 28545471

